# Effects of Different Crystallization Protocols on Marginal Gap of Lithium Disilicate Single Crowns: SEM Analysis

**DOI:** 10.3390/dj12120416

**Published:** 2024-12-18

**Authors:** Alon Shadur, Joseph Nissan, Diva Lugassy, Ariana Umansky, Eran Zenziper, Gil Ben-Izhack

**Affiliations:** 1 Department of Oral Rehabilitation, The Maurice and Gabriela Goldschleger School of Dental Medicine, Sackler Faculty of Medicine, Tel Aviv University, Tel Aviv 6997801, Israel; alonshadur@tauex.tau.ac.il (A.S.); nissandr@tauex.tau.ac.il (J.N.); arianau@mail.tau.ac.il (A.U.); ezenziper@tauex.tau.ac.il (E.Z.); 2 Department of Orthodontics, The Maurice and Gabriela Goldschleger School of Dental Medicine, Sackler Faculty of Medicine, Tel Aviv University, Tel Aviv 6997801, Israel; divaluga@mail.tau.ac.il; 3 Dental Division, Shamir (Assaf Harofeh) Medical Center, Zerifin 70300, Israel

**Keywords:** lithium disilicate, marginal gap, CAD/CAM, digital dentistry, Primescan, SEM

## Abstract

**Objective:** In everyday dentistry, lithium disilicate is a valid option for single-fix partial dentures, and this material crystallization process is available with two protocols: long and short. This study’s aim was to assess the effects of these two different crystallization protocols, long and short, on the marginal gap of lithium disilicate single crowns. **Methods:** A total of 24 abutment plastic teeth were scanned using an intra-oral scanner. For each plastic tooth, an identical pair of lithium disilicate crowns was milled (a total of 48 crowns) by a four-axis machine. Each paired sample was categorized into two groups: long crystallization (24 crowns) and short crystallization (24 crowns). To assess precision, each unit’s marginal gap (including abutments and crowns) was meticulously measured at four specified regions using a scanning electron microscope. A Kolmogorov–Smirnov test performed on the study variables indicated a normal distribution (*p* > 0.05), and it was followed by independent *t*-tests (α = 0.05). **Results:** For the long crystallization group, the mean total marginal gap values were 42.91 ± 9.67 μm, and for the short crystallization group, the values were 43.25 ± 8.14 μm, with no significant difference between the groups (*p* = 0.894). In addition, no significant differences were found between the groups regarding the mean marginal gap measurements for all four surfaces (distal (*p* = 0.310), mesial (*p* = 0.732), palatal (*p* = 0.655), and buccal (*p* = 0.535)). **Conclusions:** Both the long and short crystallization methods used for lithium disilicate single crowns demonstrated marginal gap values of less than 120 μm, which are within the clinically acceptable range, with no significant differences across any parameters between the two groups. Regarding the marginal gap value, it is recommended to use the short crystallization protocol as it is more time-efficient.

## 1. Introduction

Computer-aided design (CAD) and computer-aided manufacturing (CAM) technologies were introduced in dentistry in 1971, and today, they comprise one of the most rapidly developing fields in modern dentistry. An increasing number of chairside systems are available nowadays for dental use. Intraoral scanners have become better and faster with smaller dimensions and intuitive designs and interfaces. The combination of an intraoral scan with CAD/CAM technology has allowed for the creation of a variety of different restorations without using a physical model. There are some advantages to the digital workflow over the conventional one, such as lower costs, being less time-consuming, a variety of stable and quality materials, and patient preference [1].

The materials which are used the most for single crowns or single implant restorations are available as composite blocks, ceramic blocks with resin infiltration, or different types of ceramic blocks [2]. In the digital era, the use of these blocks is quite common in the clinic as a chair-side restoration, which can be performed in a single visit by a dentist. One of the most common materials for a single crown is lithium disilicate [3].

Among ceramic-based dental materials, lithium disilicate has established itself as a preferred choice in routine clinical practice, enjoying widespread adoption among dental practitioners. With two precursor systems (Empress 2 and IPS Emax Press; Ivoclar Vivadent, Schaan, Lichtenstein), and to accommodate the material to the needs of chairside CAD/CAM production processes, the IPS e.max CAD (Ivoclar Vivadent, Schaan, Lichtenstein) was designed for CAD/CAM technology. The material is based on partially pre-crystallized blocks which contain a mixture of 40% lithium metasilicate (Li_2_SiO_3_) crystals and lithium disilicate (Li_2_Si_2_O_5_) crystal nuclei. These blocks come in various shades and translucency levels, which are determined by the size and density of the crystals. Initially, these machinable blocks have a bluish color and moderate hardness and strength (approximately 130 MPa). This makes it easier to mill and reduces wear on machining equipment, providing clear advantages for chairside procedures [3].

After milling, the material undergoes a heat treatment process which induces full crystallization, during which the lithium metasilicates transform into lithium disilicates, comprising 70% of the final structure. After crystallization, it receives its final flexural strength of 530 MPa (according to the manufacturer). Therefore, lithium disilicate has the highest strength among glass ceramics systems, being approximately 3–4 times greater than that of other glass rich ceramics [1]. Thanks to its better translucency and a variety of shades, it can be used as a monolithic restoration or as a core material which will be veneered with apatite-containing ceramic. The indications vary from all types of single tooth or implant restorations to anterior and posterior three-unit fixed restorations [3].

As glass-ceramic prostheses crystallize, they may develop microstructural inconsistencies, which can result in unforeseen manufacturing defects, potentially compromising the accuracy and fit of the final prosthesis [4].

Studies examining the effects of crystallization on CAD/CAM-produced lithium disilicate ceramic crowns have revealed interesting findings when examining the marginal gap. When comparing the gaps in different areas of each crown before and after crystallization, researchers found that the marginal gaps increased while the internal gaps decreased. Kim et al. [5] showed that when comparing the marginal gaps of lithium disilicate crowns, before and after crystallization (only the long crystallization protocol was used), a wider gap was seen after crystallization. Gold et al. [6] also evaluated the marginal gaps of CAD/CAM-produced crowns made from lithium disilicate blocks before and after crystallization (only the long crystallization protocol was used) and found that the marginal gap for lithium disilicate crowns was significantly greater after crystallization compared with the gap before crystallization [7].

Notably, the crystallization process appears to have a significant impact on the mechanical properties of lithium disilicate, such as its strength, fracture toughness, and Vickers, as all of these variables improve. This observation holds true when comparing different CAD/CAM materials as well. These findings highlight the importance of the crystallization step in determining the final characteristics of lithium disilicate ceramic restorations [8].

Chairside systems are utilized to enhance efficiency, optimize clinical times, and provide same-day restorations. Time efficiency is a crucial factor in the restoration process. Improving the speed of fabrication while maintaining the quality of the restorations leads to a more streamlined and effective procedure [9,10].

Specialized furnaces have been developed for the post-milling crystallization heat treatment of these dental materials. These new furnaces offer faster crystallization cycles, but the impact of these accelerated processes on the final restoration quality remains uncertain. Additionally, some manufacturers have introduced compact thermal units with smaller firing chambers. These are designed to shorten the post-treatment time for materials machined in their pre-crystallized states. However, it is crucial to determine if these newer treatment methods can produce restorations with properties equivalent to those achieved with conventional units. Furnaces from the same manufacturer’s catalogue recommend using furnaces from their own product line, such as the Programat CS2 (Ivoclar-Vivadent, Schaan, Lichtenstein), for post-treatment of their materials. They offer two main crystallization programs: P1—Crystallization/Glaze—which takes approximately 24 min and has a final temperature of 840 °C (long crystallization), and P3—Speed Crystallization/Glaze Spray—which takes approximately 15 min and has the same final temperature of 840 °C (short crystallization). Each program has a maximum number of restorations to be crystallized simultaneously, while P1 can have up to six single-unit crowns and P3 can have up to two single-unit crowns at a time. For each of these firing programs, there is a recommended designated crystallization tray [11].

It is important to evaluate whether the recommended “fast” crystallization (P3) treatment suggested by the manufacture adequately achieves the desired material properties, as well as its effect on the marginal gaps of single crowns. While several studies have examined the effect of “fast” crystallization (P3) compared with regular crystallization (P1) regarding the mechanical and physicochemical properties of lithium disilicate, they did not address its effect on the marginal gaps of single crowns [8,12,13].

The accuracy of the marginal gap is crucial for the success of ceramic crown restorations. A poor marginal fit can lead to several complications: periodontal inflammation, cement breakdown, increased plaque accumulation, secondary caries, pulpal damage, and bone loss. Despite advances in technology and materials and some variation in what is considered acceptable, McLean and von Fraunhofer’s standard of 120 μm remains the widely accepted clinical threshold for a marginal gap’s width [14,15,16,17,18,19,20].

After conducting a comprehensive review of the literature, it was clear that there is a need for studies regarding the effects of different crystallization protocols (long versus short) on the marginal gap of lithium disilicate single crowns. In everyday dentistry, lithium disilicate is a valid option for single-fix partial dentures, and it is important for the clinician to know the effect of both crystallization protocols on the marginal gap of the final restoration. This study’s aim was to assess the effects of two different crystallization protocols, long and short, on the marginal gap of lithium disilicate single crowns The null hypothesis raised was that there would be no significant differences in the marginal gaps of lithium disilicate CAD/CAM restorations subjected to different crystallization protocols.

## 2. Materials and Methods

### 2.1. Study Design

In this in vitro study, two groups (*n* = 24) of lithium disilicate single crowns which underwent different crystallization protocols (long and short) were measured under scanning electron microscopy (SEM) and compared regarding the mean marginal gap values.

### 2.2. Specimen Preparation

Twenty-four standardized typodont plastic maxillary second premolar teeth (dental model: A25AN-UR51 Jacket; Nissin Dental Products INC., Kyoto, Japan) were machine-prepared by the manufacturer to ensure standardization for single crown restorations. The preparation design incorporated a 2.5 mm occlusal reduction, 6 degree total convergence angle, and 1.2 mm circumferential shoulder finish line [21].

All 24 typodont teeth were inserted into a typodont jaw and digitally scanned using a Primescan intra-oral scanner (IOS) (CEREC ^®^ Primescan; Dentsply Sirona, Milford, DE, USA). The resulting virtual models were processed using CEREC software (CEREC ^®^ SW 5.2.4, Dentsply Sirona, Milford, DE, USA). A single experienced operator (G.B.-I., with 10 years of experience) marked the finish line for all 24 digital models. The parameters established for the virtual crown design are described in Table 1 [22].

**Figure 1:**
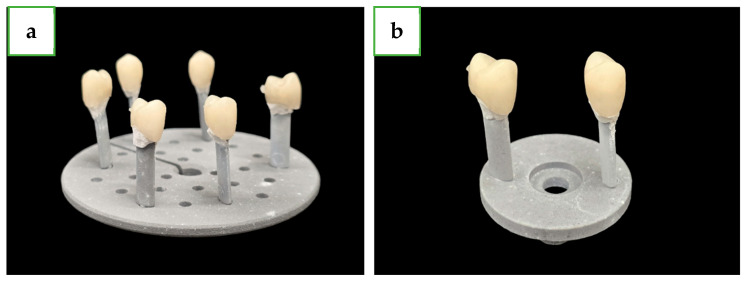
(**a**) Large crystallization tray with 6 lithium disilicate crowns after crystallization. (**b**) Small crystallization tray with 2 lithium disilicate crowns after crystallization.

For each plastic tooth, an identical pair of lithium disilicate crowns (a total of 48 crowns) were produced from lithium disilicate ceramic blocks (IPS e.max CAD; Ivoclar Vivadent, Schaan, Lichtenstein), by a 4 axis machine (CEREC MC XL; Dentsply Sirona, Milford, DE, USA) using two grits: Step Bur 12S and Cylinder Pointed Bur 12S (Dentsply Sirona, Milford, DE, USA). Each pair of crowns was then randomly divided into two groups: long crystallization (24 crowns) and short crystallization (24 crowns). Using a dedicated furnace (Programat CS2; Ivoclar Vivadent, Schaan, Lichtenstein) to fully crystallize the crowns, one group was crystallized using the long crystallization method (Table 2, P1) 6 crowns at a time, which were set on a dedicated stand, and the second group was crystallized using the short crystallization method (Table 2, P3) 2 crowns at a time, which were set on a smaller dedicated stand, as can be seen in Figure 1. The crystallization parameters are fully described in Table 2.

**Figure 2:**
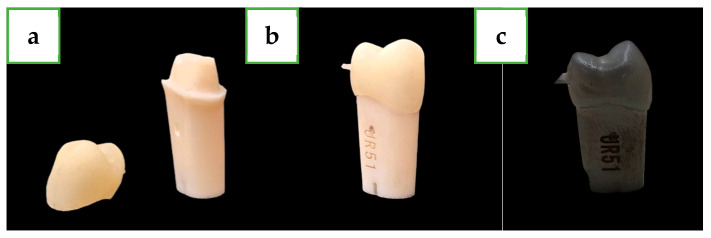
(**a**) Second maxillary premolar plastic tooth and lithium disilicate crown in the post-crystallization phase. (**b**) Crown and abutment in the post-crystallization phase before coating. (**c**) Crown and abutment after gold coating.

Crown cementation was performed using non-eugenol temporary cement (Temp-Bond™ NE™ Unidose; KaVo Kerr, Brea, CA, USA) following the manufacturer’s protocols, followed by applying finger pressure during the cement setting time as per the manufacturer’s recommendations [23]. Prior to microscopic examination, specimens were sputter-coated with gold using a mini sputter coater (SC7620; Quorum, East Sussex, UK) for 45 s to ensure conductivity (Figure 2).

### 2.3. Groups and Experimental Protocols

**Figure 3:**
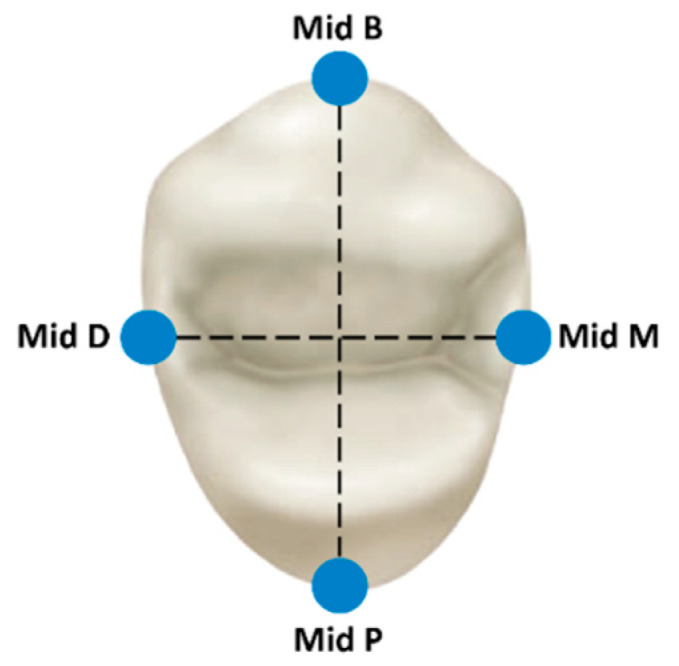
Four regions of interest for measurements at the middle of each side [24].

**Figure 4:**
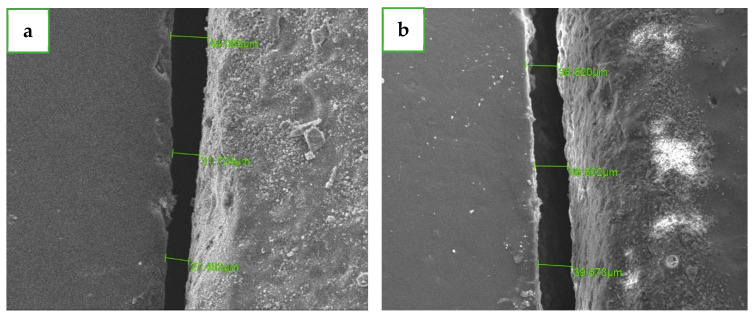
SEM micrographs showing representative marginal gap measurements from a lateral view. The mean marginal gap (MMG) was calculated from three linear measurements (green lines) perpendicular to the restoration margin. (**a**) Crown from long crystallization protocol. (**b**) Crown from short crystallization protocol. (original magnification: ×250).

All specimens were analyzed using scanning electron microscopy (SEM) (JSM-IT100; JEOL, Tokyo, Japan) at a magnification of ×250. Measurements were performed using InTouchScope™ software by a single experienced examiner (A.S.). Three vertical measurements were recorded at four reference points: buccal, mesial, palatal, and distal (Figure 3 [24]). The measurements were taken when the abutment and crown were at the most orthoradial position possible for receiving the most accurate measurements possible, yielding 12 measurements per specimen. Marginal discrepancy was measured as the vertical distance between the crown margin and the preparation finish line [25]. Following completion of the measurements for the first group, the crowns were carefully removed, and all residual temporary cement was cleaned from both crowns and abutments using a steamer (Orix, Tel-Aviv, Israel) [26]. The measurement protocol was then replicated for the second group of 24 specimens (Figure 4).

### 2.4. Main Variables and Data Analysis

The mean marginal gap (MMG) was defined and calculated as the average of the three measurements at each reference point. For each of the 24 crown and abutment units of each crystallization group, a total of 12 measurements were performed, and a total of 288 measurements were defined as the mean total marginal gap (MTMG).

A sensitivity power analysis using G*power showed that independent *t*-tests with two groups of 24 crowns would be sensitive to the effect of Cohen’s d = 0.82 with 80% power (α = 0.05, two-tailed).

Statistical analysis was performed using the Statistical Package for Social Sciences for Windows Release 23.0 (SPSS Inc., Chicago, IL, USA).

A Kolmogorov–Smirnov test was performed on the study variables, and it indicated a normal distribution (*p* > 0.05). Independent *t*-tests (α = 0.05) were used for comparing the MMGs between the long crystallization group and the short crystallization group for each surface (buccal, distal, mesial, and palatal) and for the MTMG. The statistical significance level for this work was *p* < 0.05.

## 3. Results

**Figure 5:**
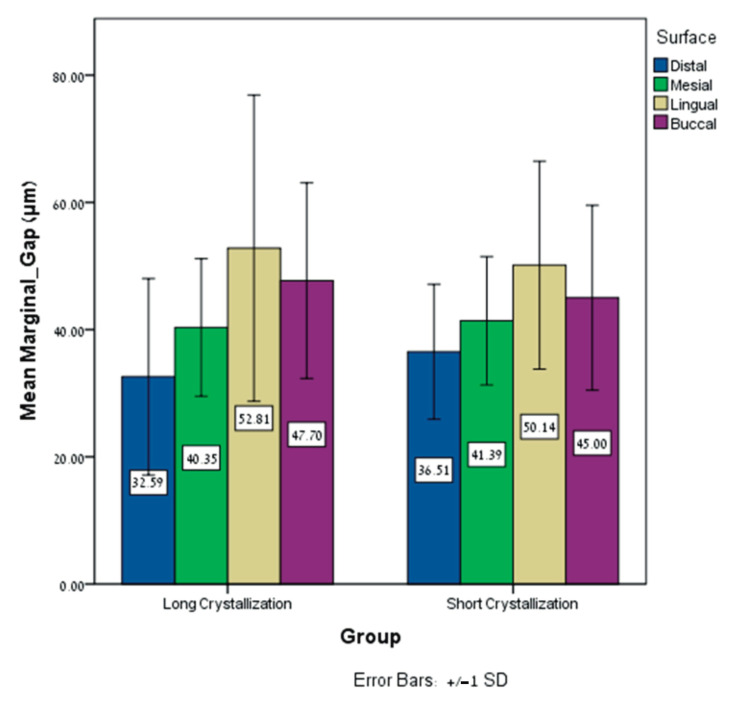
Mean ± SD of the MMG (μm) for all surfaces (distal, mesial, palatal, and buccal) for long crystallization and short crystallization groups.

The independent *t*-tests showed no significant differences regarding the mean marginal gap (MMG) between the long crystallization and short crystallization groups for all surfaces (distal (*p* = 0.310), mesial (*p* = 0.732), lingual (*p* = 0.655), and buccal (*p* = 0.535)) (Table 3, Figure 5).

The highest MMG was measured on the palatal surface of the long crystallization group (150.60 μm), and the lowest MMG was measured on the distal surface of the long crystallization group (6.74 μm).

**Figure 6:**
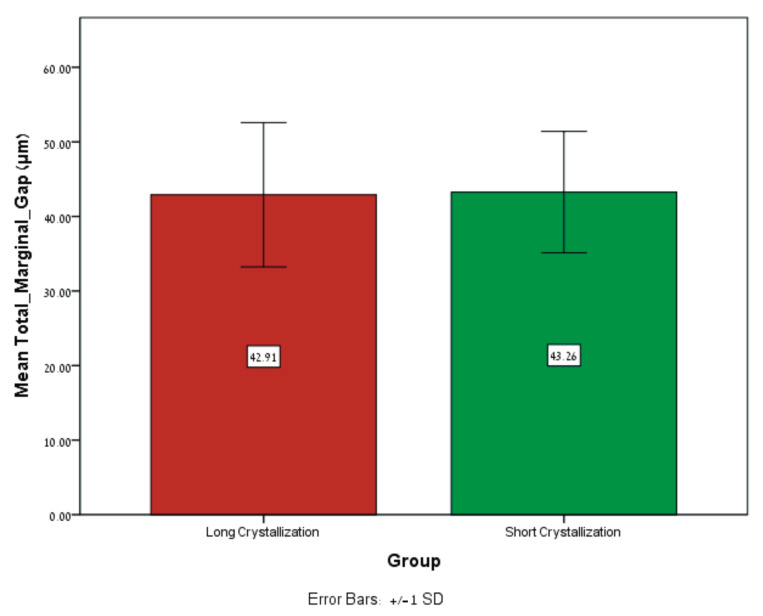
Mean ± SD of the MTMG (μm) for long crystallization and short crystallization groups.

In addition, no significant difference was found regarding the mean total marginal gap (MTMG) between the long crystallization (42.91 ± 9.67 μm) and short crystallization (43.25 ± 8.14 μm) groups (*p* = 0.894) (Table 4, Figure 6).

## 4. Discussion

The aim of this study was to assess the effects of two different crystallization protocols (long and short) on the marginal gap of lithium disilicate (IPS e.max CAD) single crowns. As the null hypothesis assumed no difference would be found between the two protocols, it had to be accepted since the results showed no significant difference.

Densification shrinkage is a well-known phenomenon which occurs during the crystallization process of glass-based materials. Previous studies have already demonstrated the effect of the crystallization process on the marginal gap of glass-ceramic restorations [5,6].

Kim et al. [5] previously examined the effect of crystallization on the marginal gap of lithium disilicate (IPS e.max CAD) single crowns produced using a CAD/CAM system. They used a CEREC Bluecam scanner (equivalent to the Primescan used in this study) and milled blocks of lithium disilicate (IPS e.max CAD HT) using a CEREC MC XL milling unit. The crystallization process was performed in a porcelain furnace (P300, Ivoclar Vivadent) at 850 °C for approximately 30 min, which is equivalent to the “long” (P1) crystallization protocol used in the current study. Kim et al. [5] used a replica technique and measured the marginal gap under a magnification of 160× with a digital microscope, reporting a mean total marginal gap of 103.12 ± 25.46 μm. In contrast, the current study used a direct measurement technique with scanning electron microscopy (SEM) at a magnification of 250× and found a mean total marginal gap of 42.91 ± 9.67 μm for the long crystallization protocol. The differences in the results can be explained using newer technologies (IOS, milling machines, and furnaces) and the higher magnification SEM measurement method in the current study.

Gold et al. [6] also examined the effect of crystallization on the marginal gap of lithium disilicate (IPS e.max CAD; Ivoclar Vivadent, Schaan, Lichtenstein) single crowns. They used a CEREC 3 system for scanning, design, and milling and a Programat CS ceramic furnace (Ivoclar Vivadent) with program 1, which is equivalent to the “long” (P1) crystallization protocol used in the current study. They measured the marginal gap using an optical microscope at a 500× magnification and reported a mean total marginal gap of 57.2 ± 16.0 μm. In comparison, the current study found a mean total marginal gap of 42.91 ± 9.67 μm for the long crystallization protocol. The differences between this study and Gold et al.’s [6] study are smaller compared with differences between this study and Kim et al.’s [5] study, which can likely be explained using a direct measurement technique and high magnification in both current study and Gold et al.’s study [6].

A more recent study by Alves et al. [27] examined the effect of crystallization on the marginal gap of lithium disilicate (IPS e.max CAD; Ivoclar Vivadent, Schaan, Lichtenstein) single crowns before (69 ± 10 μm) and after crystallization (74 ± 9 μm) using a Programat EP 5000 furnace (Ivoclar AG) with a standard crystallization protocol, which is equivalent to the P1 protocol in the current study, and they found no significant differences in the results. However, there is still a difference between the results of the current study (42.91 ± 9.67 μm for long crystallization) and those of Alves et al. [26], which can be explained by differences in the measurement techniques [28] (direct versus replica), preparation methods [29], and digital cement spacer settings [22].

In the literature, there are studies which examined the effect of post crystallization on other materials, such as zirconia lithium silicate, lithium silicate, and zirconia reinforced silicate ceramic, and for all materials, the marginal gap increased after the crystallization process [26,30].

Many studies in the current literature which examined the marginal gap or marginal fit of lithium disilicate single crowns fabricated using CAD/CAM did not specify the crystallization process used [29,31,32,33]. Alternatively, some studies utilized the long crystallization protocol [5,6,25]. As a result, direct comparisons to similar studies are not currently available. It is important that more research be conducted in this area.

Murillo-Gómez et al. [12,13] recently published studies examining the effect of a “fast” crystallization protocol (equivalent to P3 in this study) compared to a standard crystallization protocol (equivalent to P1) on the physicochemical properties of lithium disilicate CAD/CAM ceramic (IPS e.max CAD, Ivoclar-Vivadent). Their findings indicated that the “fast” crystallization protocol, both with and without glazing, resulted in weaker and less rigid structures with irregular crystals and glassy phases. While the current study found no effect on the marginal gap, these results suggest that the mechanical and physicochemical properties of lithium disilicate may be impacted by the crystallization protocol. Further studies examining all relevant parameters together are needed to fully understand the effects of “fast” crystallization on lithium disilicate.

Some limitations of the current study must be acknowledged. It was an in vitro study, only one type of lithium disilicate was examined, a single measurement technique was used, and only one type of furnace was employed. The results are therefore limited to single crowns.

Additional research is required to better understand the influence of different crystallization protocols on the marginal gap of lithium disilicate single crowns, as the authors are unaware of other similar studies which assessed the marginal gap of lithium disilicate single crowns using different crystallization protocols. Factors which should be investigated include the digital settings (radial spacer and occlusal spacer), preparation parameters (convergence and finish line), the use of different furnaces, and the evaluation of various types of lithium disilicate materials.

## 5. Conclusions

Within the limitations of this in vitro study, it was found that both the long and short crystallization protocols for lithium disilicate single crowns produced clinically acceptable marginal gaps (<120 μm), with no significant differences between the two protocols. Given its time efficiency, the short protocol is recommended.

## Figures and Tables

**Table 1:** Crowns design parameters used in this study.

Internal Relief	Contact Strength Parameters	Material Thickness Parameters	Marginal Parameters
Radial spacer: 90 μm	Proximal contacts: 25 μm	Radial minimal thickness: 1000 μm	Margin ramp width: 50 μm
Occlusal spacer: 120 μm	Dynamic contacts: 25 μm	Occlusal minimal thickness: 1500 μm	Margin thickness: 50 μm
	Occlusal contacts: 25 μm		Margin ramp angle: 60°

**Table 2:** Programat CS2 crystallization and firing parameters for each protocol: long and short.

Features or Protocol	Long P1 (Approx. 24 min)	Short P3 (Approx. 15 min)
Standby temperature	403 °C	403 °C
Closing time	6:00 min:s	1:30 min:s
Heating rate	90 °C/min	90 °C/min
Firing temp 1	820 °C	820 °C
Holding time	0:10 min:s	0:10 min:s
Heating rate to temp 2	30 °C/min	30 °C/min
Firing temp 2	840 °C	840 °C
Holding time 2	7:00 min:s	7:00 min:s
Vacuum 1	550–820 °C	550–820 °C
Vacuum 2	820–840 °C	820–840 °C
Long-term cooling	700 °C	700 °C
Cooling rate	0 °C/min	0 °C/min

**Table 3:** Mean ± SD, range, and minimum and maximum MMG (μm) for all surfaces (distal, mesial, palatal, and buccal) for long crystallization and short crystallization groups (α = 0.05).

	Distal Surface			Mesial Surface			Palatal Surface			Buccal Surface		
Mean Marginal Gap (MMG) (μm)	Mean ± SD	Range	Min Max	Mean ± SD	Range	Min Max	Mean ± SD	Range	Min Max	Mean ± SD	Range	Min Max
Long crystallization	32.58 ± 15.43	64.12	6.74 70.86	40.34 ± 10.8	41.60	20.67 62.27	52.80 ± 24.07	124.95	25.65 150.60	47.70 ± 15.39	55.36	20.40 75.76
Short crystallization	36.50 ± 10.60	37.26	18.42 55.68	41.38 ± 10.09	36.93	25.04 61.97	50.13 ± 16.33	53.55	23.26 76.81	45.00 ± 14.53	49.19	24.71 73.89

**Table 4:** Mean ± SD, range, and minimum and maximum of the MTMG (μm) for long crystallization and short crystallization groups (α = 0.05).

Mean Total Marginal Gap (MTMG) (μm)	Mean ± SD	Range	Min Max
Long Crystallization	42.91 ± 9.67	43.74	27.04 70.78
Short Crystallization	43.25 ± 8.14	28.20	29.42 57.62

## Data Availability

The original contributions presented in this study are included in the article. Further inquiries can be directed to the corresponding author.
